# Antimicrobial Susceptibility of *Mycoplasma bovis* Isolates from Veal, Dairy and Beef Herds

**DOI:** 10.3390/antibiotics9120882

**Published:** 2020-12-09

**Authors:** Jade Bokma, Linde Gille, Koen De Bleecker, Jozefien Callens, Freddy Haesebrouck, Bart Pardon, Filip Boyen

**Affiliations:** 1 Department of Large Animal Internal Medicine, Faculty of Veterinary Medicine, Ghent University, Salisburylaan 133, 9820 Merelbeke, Belgium; linde.gille@uliege.be (L.G.); bart.pardon@ugent.be (B.P.); 2 Department of Pathology, Bacteriology and Avian Diseases, Faculty of Veterinary Medicine, Ghent University, Salisburylaan 133, 9820 Merelbeke, Belgium; freddy.haesebrouck@ugent.be (F.H.); filip.boyen@ugent.be (F.B.); 3 Animal Health Service-Flanders, Industrielaan 29, 8820 Torhout, Belgium; koen.debleecker@dgz.be (K.D.B.); jozefien.callens@dgz.be (J.C.)

**Keywords:** epidemiological cut-off methods, gamithromycin, genomic clusters, iterative statistical method, normalized resistance interpretation, visual estimation

## Abstract

*Mycoplasma bovis* is an important pathogen causing mostly pneumonia in calves and mastitis in dairy cattle. In the absence of an effective vaccine, antimicrobial therapy remains the main control measure. Antimicrobial use in veal calves is substantially higher than in conventional herds, but whether veal calves also harbor more resistant *M. bovis* strains is currently unknown. Therefore, we compared antimicrobial susceptibility test results of *M. bovis* isolates from different cattle sectors and genomic clusters. The minimum inhibitory concentration of nine antimicrobials was determined for 141 Belgian *M. bovis* isolates (29 dairy, 69 beef, 12 mixed, 31 veal farms), and was used to estimate the epidemiological cut-off. Acquired resistance was frequently observed for the macrolides, while no acquired resistance to oxytetracycline and doxycycline, minimal acquired resistance to florfenicol and tiamulin, and a limited acquired resistance to enrofloxacin was seen. *M. bovis* isolates from beef cattle or genomic cluster III had higher odds of being gamithromycin-resistant than those from dairy cattle or genomic clusters IV and V. In this study, no cattle industry could be identified as source of resistant *M. bovis* strains. A single guideline for antimicrobial use for *M. bovis* infections, with a small remark for gamithromycin, is likely sufficient.

## 1. Introduction

In the last decade, *Mycoplasma bovis (M. bovis)* has come to the forefront as an economically important bacterium with a large impact on health, welfare and antimicrobial use (AMU) in cattle operations worldwide [1]. The bacterium is mainly feared as the cause of pneumonia, arthritis and otitis in calves, and pneumonia and mastitis in adult cattle [2,3]. In the absence of an effective vaccine, antimicrobial therapy remains a crucial factor to control an outbreak.

In recent years, a decrease in the antimicrobial susceptibility of *M. bovis* to various antimicrobial classes targeting protein synthesis (e.g., phenicols, tetracyclines, lincosamides and macrolides) and DNA synthesis (e.g., fluoroquinolones) has been reported in different countries [4,5,6,7,8,9]. Especially for macrolides, high percentages of resistant *M. bovis* isolates are reported, while fluoroquinolones remain the most effective antimicrobial in vitro in most countries [5,9,10,11], except for Spain and Italy [6,7]. Geographical differences in the antimicrobial susceptibility of *M. bovis,* as well as differences between strains isolated from various predilection sites, such as the joint, udder and lung, have been described [5,10,12]. Additionally, more antimicrobial resistance was observed in Canadian *M. bovis* strains obtained from dead animals compared to those isolated from healthy animals [8]. Despite the fact that antimicrobial susceptibility differences between production systems have been shown for other respiratory pathogens, such as *Pasteurella* and *Mannheimia* isolates [13], this was not previously explored for *M. bovis*. The quantity and quality of AMU can differ greatly between production systems, with veal calf operations showing much higher AMU compared to conventional herds [14]. Varying AMU may result in a different resistance selection pressure, subsequently changing antimicrobial susceptibility patterns or supporting the clonal emergence of specific *M. bovis* strains in outbreaks [7,9,15]. If production-specific antimicrobial susceptibility exists for *M. bovis*, it might be necessary to adjust *M. bovis* treatment guidelines to specific production systems. Additionally, whether antimicrobial resistance is associated with specific genetic strains is not clear, as some studies observed no association between genetic subtypes based on the *polC* subtyping scheme [11], while others did for lincosamides and macrolides [9]. With whole genome sequencing (WGS) becoming more popular and commercially available for identification and strain typing [16,17,18,19], it might be helpful to determine whether phenotypic antimicrobial susceptibility patterns are associated with genomic clusters of *M. bovis*. Therefore, the objective of the present study was to compare the antimicrobial susceptibility results of *M. bovis* isolates obtained from veal calf, conventional dairy and beef herds, and to explore the association of antimicrobial resistance (AMR) with specific *M. bovis* genomic clusters. 

## 2. Results

### 2.1. Antimicrobial Susceptibility of Belgian M. bovis 

The minimum inhibitory concentration (MIC) results of 141 epidemiologically unrelated *M. bovis* isolates are shown in Table 1. All obtained isolates were identified as *M. bovis* with MALDI-TOF MS (score value ≥1.7). The MIC values for the *M. bovis* PG45 reference strain were within a small range of dilutions between runs for gamithromycin (8–16 µg/mL), tilmicosin (0.12–0.5 µg/mL), florfenicol (1–2 µg/mL), doxycycline (≤0.06–0.12 µg/mL), enrofloxacin (≤0.06–0.12 µg/mL), tylosin (≤0.06–0.12 µg/mL), tiamulin (≤0.03 µg/mL), and oxytetracycline (≤0.12 µg/mL), and were similar to previously described results [7,10,12,20,21,22]. The quality control (QC) MIC values for *Staphylococcus aureus* subsp. aureus (ATCC^®^29213^TM^) and *Escherichia coli* (ATCC^®^25922^TM^) were within the acceptable QC ranges, as provided by the Sensititre manufacturer. Results for gentamicin were excluded from this study, as the quality control was not passed, and the MIC values for the type strain *M. bovis* PG45 deviated from previous studies [12,22]. However, no QC strains were included in previous studies, and therefore the results are included in Supplementary File 1.

### 2.2. Interpretation of MIC Values

The epidemiological cut-off values (ECOFF), as determined using the different methods, and the percentages of isolates belonging to the wild type and non-wild type population, are shown in Table 2. For all three ECOFF methods, acquired resistance was frequently observed for macrolides (gamithromycin, tylosin), while only a few isolates showed acquired resistance against florfenicol, enrofloxacin and tiamulin. Following the visual estimation method, no acquired resistance against oxytetracycline and doxycycline was observed, although the statistical methods categorized part of the population (3.6–13.0%) as non-wild type for doxycycline.

We were able to determine the ECOFF for seven out of the eight antimicrobials with the visual estimation method, whereas normalized resistance interpretation (NRI) and ECOFFinder determined a reliable ECOFF in eight out of eight and three out of eight of the antimicrobials, respectively. The NRI method was able to determine an ECOFF for every antimicrobial tested, even when this needed extrapolation from the tested MIC range, such as for tilmicosin. However, when the standard deviation of the normal distribution of the wild type MIC value is >1.2 log_2_, the program provides only the “putative wild type population”, as was the case for the macrolides. The ECOFFinder method was only able to determine reliable results (good fit plots for residuals) for florfenicol and tetracyclines. Truncated distributions influence the reliability or possibility of interpreting some of the results, such as those for the macrolides. All three methods determined the ECOFF values for the corresponding antimicrobials within one dilution, except for doxycycline and tiamulin. This results in substantial differences in the non-wild type population. When ECOFFinder 95% was used for doxycycline, 13% was categorized as non-wild type in comparison to 0% when using the visual estimation method. For tiamulin, the visual method indicated 0.7% non-wild type, whereas NRI indicated 15.1%. This might be due to the combination of “tailing” and the lack of a normal distribution, which complicates the interpretation of the MIC distributions, both visually and statistically.

### 2.3. Variability of Antimicrobial Susceptiblity Per Production System

The distribution of *M. bovis’* MIC values for the different antimicrobials and per production system are available in Supplementary File 2. Logistic regression did not show significant differences in antimicrobial resistance between production systems, except for gamithromycin (Table 3). Beef *M. bovis* isolates (58.21% acquired resistance) had three-times higher odds (CI 95%: 1.23–7.69) of gamithromycin resistance than dairy isolates (31.03%; *p* = 0.02).

The MIC_50_ and MIC_90_ values are shown per sector in Table 4. No differences in MIC_50_ were observed between sectors for tilmicosin, doxycycline, and tiamulin. A single two-fold dilution difference in MIC_50_ between herd types was observed for florfenicol (highest in veal), oxytetracycline (lowest in dairy) and enrofloxacin (lowest in beef). A difference between herds was seen for gamithromycin and to a lesser extend for tylosin. The MIC_50_ of gamithromycin was higher in beef cattle (>128 µg/mL) than in dairy (16 µg/mL) or veal calf isolates (32 µg/mL). No difference was observed in MIC_90_ for florfenicol, tilmicosin, and gamithromycin, while a single two-fold dilution was observed in MIC_90_ for oxytetracycline (lowest in dairy), doxycycline (highest in beef), tylosin (lowest in dairy), tiamulin (lowest in veal) and enrofloxacin (highest in dairy). 

### 2.4. Association between AMR and Genomic Cluster 

**Figure 1:**
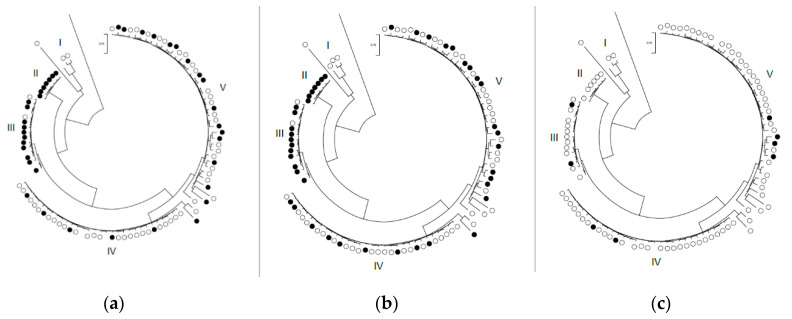
Phylogenetic tree with the distribution of Belgian *M. bovis* isolates being susceptible (○) or resistant (●) for gamithromycin (**a**), tylosin (**b**), and enrofloxacin (**c**) based on the visual estimation method. These isolates were classified into five genomic clusters by an SNP-analysis of 100 Belgian *M. bovis* isolates [18]. The figure was created using MEGA-X software with *M. bovis* isolates obtained between 2014–2019.

In Figure 1, the distributions of the wild type and non-wild type *M. bovis* isolates for gamithromycin, tylosin, and enrofloxacin are shown over the five clusters obtained by whole genome sequencing. Most of the macrolide-resistant isolates were located in clusters II and III, whereas cluster I contained isolates susceptible to all antimicrobials. Statistical analysis showed that *M. bovis* isolates from cluster III (85% acquired resistance) had 22.7- (CI95%: 4.0–125.0, *p* < 0.01) and 7.9 (CI95%: 1.5–40.0, *p* = 0.01) -fold higher odds for gamithromycin resistance compared to clusters IV (19%) and V (41%), respectively. No significant association was found for tylosin or enrofloxacin. 

## 3. Discussion

In this study, the susceptibility of 141 *M. bovis* isolates obtained from beef, dairy and veal calves was tested against nine antimicrobial agents covering the 6 antimicrobial classes most frequently used to control *M. bovis* in Belgium, though the results of gentamicin (covering the aminoglycosides) were excluded. Since neither clinical breakpoints (CBPs) nor guidelines to interpret ECOFFs for *M. bovis* are available, three methods (visual, NRI and ECOFFinder) to determine ECOFF and interpret the antimicrobial susceptibility of *M. bovis* were explored. 

Although one should be cautious in comparing the results of different studies, because of the lack of internationally recognized standard protocols, the observed MIC values and distributions in this study were similar to previous publications from Western Europe [6,10,23,24]. A large number of isolates had high MIC values for macrolides, resulting in more than half of the isolates being non-wild type for gamithromycin or tylosin. Cross-resistance between tylosin and tilmicosin has been described for *M. bovis* [25,26], but determination of ECOFF based on the MIC values for tilmicosin was not possible, as no normal distribution was obtained due to the very high MIC values. 

Surprisingly, there were no indications of acquired resistance against tetracyclines. The current study showed the decreased MIC_50–90_ values (1–4 µg/mL) of *M. bovis* for oxytetracycline compared with a 20-year-old Belgian study reporting MIC_50–90_ values of 2 and 32 µg/mL, respectively [23]. Additionally, in other European countries, a similar trend has been observed in recent years [6,10]. For doxycycline, the percentages of acquired resistance depended on the ECOFF method used (0% to 13%). When using the 95% rule with ECOFFinder, 13% acquired resistance was obtained, while when using the 99% rule only 3.6% resistant isolates were observed. One should be aware that the decision of using 95% or 99% can influence the outcome by increasing either the sensitivity for non-wild type (95%) or the specificity for the wild type population (99%). The distribution and MIC_50–90_ for florfenicol were similar (2–8 µg/mL) to previously published data [4,6,10], and only four isolates showed acquired resistance (MIC 32 µg/mL). In general, a small non-wild type population was observed for tiamulin. In Europe, pleuromutilins are not registered for use in cattle, while this class of antimicrobials is registered for the treatment of *Mycoplasma* infections in pigs and poultry. In addition, valnemulin appears to be very effective against *M. bovis* in vivo [27], and low numbers of acquired resistance have been reported in *M. bovis* isolates in France, Spain, and Hungary as well [4,7,26]. One possible non-wild type isolate for tiamulin was identified in this study. In a previous study, all tiamulin-resistant mutant strains showed cross-resistance against florfenicol [26], which was not observed in the current study. The distribution and MIC_50–90_ values for enrofloxacin (0.5–1 µg/mL) were comparable with previous studies [4,10,23], except for that of Klein et al. (2019), which found higher MIC_90_ values (8 µg/mL). 

Gentamicin did not pass the QC with *S. aureus* (ATCC^®^29213^TM^) and *E. coli* (ATCC^®^25922^TM^), and also the MIC for *M. bovis* PG45 deviated from previous studies [12,22]; based on these observations the results were excluded. Previous studies did not include these quality control strains, and therefore we are not aware of whether this is a reoccurring problem [12,22,23]. It is likely that specific medium components, resulting in an adjusted pH, have altered the results of the quality control strains [28], but as there is no standard protocol or quality control standard for *M. bovis* PG45, more research is necessary. Notwithstanding this, we included the results in the supplementary data to contribute to this research (Supplementary File 1).

We did not observe significant sector-specific antimicrobial resistance, except for with gamithromycin. *M. bovis* isolates from beef cattle had higher odds of being non-wild type than those from dairy cattle. Dairy cattle isolates also had the lowest MIC_50_ values for tylosin. This could possibly be explained by the non-registration of macrolides for use in lactating animals, and the high use of macrolides to combat bovine respiratory disease in beef cattle and veal calves. Additionally, other factors, such as age, housing conditions and milk diet, could play a part in the evolution of antimicrobial resistance in different production systems [29]. Considering the small difference in AMR over sectors, together with previously obtained knowledge of the lack of sector-specific *M. bovis* strains in Belgium [18], a single guideline for the antimicrobial use for *M. bovis* infections covering all different cattle sectors in Belgium, with a small remark for gamithromycin, is likely sufficient. 

All used methods to determine the ECOFF are in some way based on a normal distribution. As a consequence, problems occurred with truncated MIC distributions (e.g., tilmicosin, tiamulin). Although the NRI method was able to determine more ECOFFs than the ECOFFinder (only florfenicol and tetracyclines) in an objective manner, the visual estimation method was mostly in agreement with these methods. Even though it was more subjective, the visual estimation method has the advantage that expert opinion and additional information from MIC data obtained from other class representatives or previous reports can be taken into account. For instance, even though a bimodal distribution was observed for tylosin, the population showing the lower MIC values might not represent the true wild type population. This is supported by a previous MIC study showing a much lower ECOFF (2 µg/mL), while similar QC values were obtained [30]. In addition, it has been previously shown that the specific mutations associated with macrolide resistance were absent in isolates with MIC values between <0.5 and 4 µg/mL [25]. Therefore, an overall shift from *M. bovis* wild type to non-wild type for tylosin is suspected. The same line of reasoning is applicable to tilmicosin. Lerner et al. (2014) did not find any mutations associated with macrolide resistance in isolates with tilmicosin MIC values between <0.5 and 32 µg/mL. Therefore, it seems that all the isolates in this study acquired resistance to tilmicosin to some extent, except for one isolate with an MIC of 1 µg/mL for tylosin and 8 µg/mL for tilmicosin, probably representing the wild type population for 16-ring macrolides. Indeed, a recent study showed very high MIC values (≥256 µg/mL) for >80% of the *M. bovis* population against tilmicosin [10], whereas an older study showed a large population with lower MIC values between 0.5 and 32 µg/mL [21]. 

Finally, we observed an association between gamithromycin susceptibility patterns and previously published genomic clusters. *M. bovis* isolates in clusters II and III were more frequently belonging to the non-wild type population than those in clusters IV and V. This might be due to the higher heterogeneity in clusters IV and V, caused by genetic drift [18]. Yet, we should be careful in our conclusions, as we are not aware of the influence of genetic drift within any of the clusters on antimicrobial susceptibility data. We feel on this point that even when strain typing can be done very fast and easily, this should always be supplemented with phenotypic antimicrobial susceptibility testing (AST) to detect acquired resistance in *M. bovis* outbreaks. Nevertheless, strain typing could support the surveillance of AMR by pointing out whether isolates are clonally spread or (closely) related to each other. 

## 4. Materials and Methods

### 4.1. Mycoplasma Bovis Collection

One hundred forty-one epidemiologically independent *M. bovis* isolates, originating from 29 dairy, 69 beef, 12 mixed (both dairy and beef) and 31 veal farms, were included in this study. Isolates were obtained from the respiratory tract (128), middle ear (4), milk (5), joint (2), abscess (1) and seroma (1), collected in Belgium between 2016 and 2019, with the exception of one isolate which was obtained in 2014. One hundred of these isolates have been strain-typed previously [18]. All isolates were obtained from diagnostic samples collected by field veterinarians from clinical cases, in compliance with the EU legislation on ethics in animal experimentation [2010/63/EU]. All samples were cultured on a modified pleuropneumonia-like organism (PPLO) agar plate and incubated for 7–10 days (37 °C, 5% CO_2_). Presumptive *M. bovis* identification was based on the typical fried-egg colony appearance on modified PPLO agar and the presence of lipase activity as tested on medium containing Tween-80 [31]. Final identification was performed with MALDI-TOF MS as described before [32]. All samples were stored at −80 °C until further analysis.

### 4.2. Antimicrobial Susceptibility Testing

MICs were obtained following the guidelines for the MIC testing of veterinary *Mycoplasma* spp., described by Hannan [33]. *M. bovis* isolates were thawed and cultured on modified PPLO agar. After 7 days of incubation at 37 °C in a 5% CO_2_-enriched atmosphere, colonies were inoculated in modified PPLO broth (pH 7.8) (Difco^TM^, BD Diagnostic Systems, Sparks, Md.) supplemented with 25% inactivated horse serum (Gibco^TM^), 0.7% technical yeast extract, 0.5% sodium pyruvate (ReagentPlus, Sigma-Aldrich, Overijse, Belgium), 0.5% d-(+)-glucose monohydrate (Sigma-Aldrich, Overijse, Belgium) and 0.005% phenol red as growth indicator. After 3 days of incubation (37 °C; 5% CO_2_), a bacterial suspension of approximately 10^8^ CFU/mL was obtained. Ten-fold serial dilutions were made using the same broth, and 200 µL of the diluted suspension with approximately 10^5^ CFU/mL was transferred to each well of a custom-made 96-U-bottom-well Sensititre microplate (Thermofisher) containing doubling florfenicol concentrations at between 0.25 and 128 µg/mL, oxytetracycline (0.12–128 µg/mL), doxycycline (0.06–32 µg/mL), tilmicosin (0.06–128 µg/mL), tylosin (0.06–32 µg/mL), gamithromycin (0.06–256 µg/mL), tiamulin (0.03–1 µg/mL), gentamicin (0.06–32 µg/mL) and enrofloxacin (0.06–32 µg/mL). Additionally, two growth control wells (no antimicrobial, with inoculum), a sterility control well (200 µL uninoculated broth, pH 7.8) and a pH control well (200 µL uninoculated broth, pH 6.8) were added to each plate. A quantity of 100 µL from one growth control well was used to perform ten-fold dilutions and subsequent inoculation on a modified PPLO agar plate. Colonies were counted after 7 days of incubation at 37 °C in a 5% CO_2_-enriched atmosphere, to confirm that inoculum concentrations were within a 10^3^–10^5^ CFU/mL range.

Plates were sealed with adhesive foil and incubated at 37 °C. The interpretation of color change from red to orange/yellow was done after 48 and 72 h of incubation. The lowest antimicrobial concentration without color change was recorded as the MIC at the earliest time point at which the growth control well had the same color as the pH control well. When for a certain isolate, more than one skipped well was observed, the results for these isolates were excluded. When only one well was skipped, the highest MIC value was listed [34]. Quality control was performed in every run (six in total) by determining the MIC values of the *M. bovis* strain PG45 (ATCC 25523) and comparing these to previously published values [7,12,20,22]. Reference strains *Staphylococcus aureus* ATCC^®^29213^TM^ and *Escherichia coli* ATCC^®^25922^TM^ were included as extra QC strains in the same broth as *M. bovis*, but were observed after 24 h of incubation.

### 4.3. Interpretation of MIC Values

Due to the lack of clinical breakpoints (CBPs), the interpretation of the MIC values of *M. bovis* is not straightforward [3,21,35]. The best option for interpreting the *M. bovis* MIC data is probably to determine ECOFF. With this method, wild type bacterial populations are distinguished from those with acquired resistance (non-wild type) by observing the MIC distribution. Three methods to estimate the ECOFF were compared in this study—the visual estimation (“eyeball”) method based on the uni-, bi- or multimodal MIC distribution and/or tailing, as described previously [35,36], as well as two statistical methods, “Normalized Resistance Interpretation (NRI)” (http://www.bioscand.se/nri/, Bioscand AB, Täby, Sweden; [37]) and the “Iterative Statistical Method” processed in ECOFFinder (version 2.1; https://www.eucast.org/mic_distributions_and_ecoffs/, EUCAST) [38,39]. Instructions provided by the founders were followed. When using the NRI method, standard deviations of the normal distribution of wild type MIC values exceeding 1.2 log_2_ result in a tentative estimate of the ECOFF, and one can only speak of the “putative wild type group”. With ECOFFinder, plots for residuals were checked and categorized, corresponding to whether the residuals scattered on either side of the horizontal axis in the center (well fit, selected subset values are considered reliable), only partly (poor fit) or not at all (no fit). As users can choose the cut-off value (95% to 99.9%) with ECOFFinder, depending on the intended use and influencing the sensitivity and specificity of the (non-)wild type population, both the 95% and 99% cut-offs were determined. In addition, the MIC_50_ and MIC_90_ were calculated as the lowest MIC at which at least 50% and 90% of the isolates in a test population are inhibited in their growth, respectively. Since no ECOFF within the testing range of our study could be obtained by any of the three methods for tilmicosin, previously published data and cross-resistance with tylosin were taken into account to make the decision that the isolates with MIC ≥32 µg/mL belonged to the non-wild type population (see discussion section). The latter tilmicosin ECOFF, together with the ECOFFs obtained with the visual estimation method, were used in further analysis to compare the AMR in *M. bovis* isolates obtained from different cattle sectors or belonging to specific genomic clusters. 

### 4.4. Statistical Analysis

To determine whether there are significant differences between conventional herds (dairy, beef) and veal calves, a logistic regression was performed on binary variables, representing acquired resistance (1) and wild type (0) isolates. A *p*-value smaller than 0.05 was considered statistically significant. The Hosmer–Lemeshow test was included to determine the goodness of fit of the model (SPSS Statistics 26). To allow a meaningful statistical analysis, only for the antimicrobials for which 5 to 95% acquired resistance was observed was statistical analysis done.

In a former study, a subset of 100 isolates of the currently used *M. bovis* database had been strain-typed, as described earlier [18]. These isolates have been categorized into 5 phylogenetic clusters, based on single nucleotide polymorphism (SNP) analysis [18]. In the present study, the association between the presence of acquired resistance according to the visual estimation method and the phylogenetic clusters of this subset of strains was investigated for antimicrobials for which 5 to 95% acquired resistance was observed, and further visualized in MEGA-X [40]. Logistic regression on binary variables (1: acquired resistance; 0: wild type) was only performed for clusters III to V, as clusters I and II did not contain enough *M. bovis* isolates for the model to run. 

## 5. Conclusions

The high acquired resistance percentages of *M. bovis* in Belgium were observed for macrolides, with almost all isolates having acquired a resistance to 16-ring macrolides and a large proportion to 15-ring macrolides. In addition, a minimal acquired resistance to florfenicol and tiamulin was observed, a limited acquired resistance to enrofloxacin, and almost no resistance to the tetracyclines. A higher AMR for gamithromycin was observed in beef cattle compared to dairy, but the veal industry could not be identified as a reservoir of resistant *M. bovis* strains. A single guideline for the antimicrobial use of *M. bovis* infections covering all different cattle sectors in Belgium, with a small remark for gamithromycin, is likely sufficient. In addition, only *M. bovis* strains belonging to clusters II and III had more isolates with acquired resistance for gamithromycin compared to IV and V. Therefore, this study shows that strain-typing cannot replace the phenotypic AST of *M. bovis* in surveillance programs.

## Figures and Tables

**Table 1:** Distribution of minimum inhibitory concentration (MIC) values (µg/mL) of 141 *M. bovis* isolates obtained from cattle in Belgium between 2016–2019.

Class	Antimicrobial	Distribution of MICs (µg/mL)														
		≤0.03	≤0.06	0.12	0.25	0.5	1	2	4	8	16	32	64	128	>128	Total
Phenicol	Florfenicol	ND	ND	ND	1	5	27	44	43	13	3	4			ND	140
Tetracycline	Oxytetracycline	ND	ND	5	2	15	49	46	17	5					ND	139
	Doxycycline	ND	3	4	43	47	23	13	5				ND	ND	ND	138
Macrolide	Tilmicosin	ND								1			3	23	114	141
	Tylosin	ND					1	3	11	16	22	7	77 ^a^	ND	ND	137
	Gamithromycin	ND	1				3	2	8	19	26	9	1	4	66	139
Pleuromutilin	Tiamulin	46	43 ^c^	29	15	5			1 ^b^	ND	ND	ND	ND	ND	ND	139
Fluoroquinolone	Enrofloxacin	ND	1	17	51	37	20	2	3	2	2	1	1 ^a^	ND	ND	137

ND: not determined, ^a^ MIC ≥ 32, ^b^ MIC ≥ 2, ^c^ MIC 0.06; ECOFFs based on the visual estimation method are shown as black vertical lines.

**Table 2:** Epidemiological cut-offs for *M. bovis* from Belgian cattle (*n* = 141) based on the visual estimation method, normalized resistance interpretation (NRI) and with ECOFFinder, resulting in different percentages of wild type (WT) and non-wild type (n-WT).

Class	Antimicrobial	Visual Estimation	WT (%)	n-WT (%)	NRI	WT (%)	n-WT (%)	ECOFFinder (95%/99%) *	WT (%)	n-WT (%)
Phenicol	Florfenicol	>16	97.1	2.9	>16	97.1	2.9	>8/16 (+)	95.0/97.1	5.0/2.9
Tetracycline	Oxytetracycline	>8	100.0	0.0	>8	100.0	0.0	>4/8 (+)	96.4/100.0	3.6/0.0
	Doxycycline	>4	100.0	0.0	>2	96.4	3.6	>1/2 (+)	87.0/96.4	13.0/3.6
Macrolide	Tilmicosin	ND	-	-	>1024	-	-	ND	-	-
	Tylosin	>32	43.8	56.2	>128 #	-	-	ND	-	-
	Gamithromycin	>64	49.6	50.4	>128 #	52.5	47.5	ND	-	-
Pleuromutilin	Tiamulin	>0.5	99.3	0.7	>0.125	84.9	15.1	>0.06/0.06 (−)	-	-
Fluoroquinolone	Enrofloxacin	>2	93.4	6.6	>1	92.0	8.0	>1/2 (±)	92.0/93.4	8.0/6.6

# = tentative estimate, as standard deviation >1.2 log_2_. * plots for residuals were checked and categorized in either well fit (+), poor fit (±) or no fit (−) corresponding to whether the subset values are reliable or not. ND: not possible to determine.

**Table 3:** Results of logistic regression of antimicrobial resistant *M. bovis* isolates obtained from beef, dairy and veal calves between 2016–2019 in Belgium.

Antimicrobial	Sector	ECOFF	WT (%)	n-WT (%)	OR	CI95%	*p*-Value
Florfenicol	Beef	>16	95.59	4.41			
	Dairy	>16	96.55	3.45			
	Veal	>16	100.00	0.00			
Oxytetracycline	Beef	>8	100.00	0.00			
	Dairy	>8	100.00	0.00			
	Veal	>8	100.00	0.00			
Doxycycline	Beef	>4	100.00	0.00			
	Dairy	>4	100.00	0.00			
	Veal	>4	100.00	0.00			
Tylosin	Beef	>32	42.42	57.58	Ref		0.48
	Dairy	>32	51.62	48.38	0.60	(0.25–1.44)	0.25
	Veal	>32	41.94	58.06	1.02	(0.43–2.42)	0.96
Tilmicosin	Beef	>32	1.45	98.55			
	Dairy	>32	0.00	100.00			
	Veal	>32	0.00	100.00			
Gamithromycin	Beef	>64	41.79	58.21	Ref		0.06
	Dairy	>64	68.97	31.03	0.32	(0.13–0.81)	0.02
	Veal	>64	51.61	48.39	0.67	(0.29–1.58)	0.36
Tiamulin	Beef	>0.5	98.53	1.47			
	Dairy	>0.5	100.00	0.00			
	Veal	>0.5	100.00	0.00			
Enrofloxacin	Beef	>2	92.54	7.46	Ref		0.98
	Dairy	>2	92.59	7.41	0.99	(0.18–5.45)	0.99
	Veal	>2	93.55	6.45	0.86	(0.16–4.67)	0.86

OR = odds ratio; CI95%: 95% confidence interval.

**Table 4:** MIC_50_ and MIC_90_ (µg/mL) of all *M. bovis* isolates and per sector, obtained from cattle in Belgium between 2016–2019.

Class	Antimicrobial	Total (*n* = 144)		Beef (*n* = 70)		Dairy (*n* = 31)		Veal (*n* = 32)	
		MIC_50_	MIC_90_	MIC_50_	MIC_90_	MIC_50_	MIC_90_	MIC_50_	MIC_90_
Phenicol	Florfenicol	2	8	2	8	2	8	4	8
Tetracycline	Oxytetracycline	1	4	2	4	1	2	2	4
	Doxycycline	0.5	2	0.5	2	0.5	1	0.5	1
Macrolide	Tilmicosin	>128	>128	>128	>128	>128	>128	>128	>128
	Tylosin	>32	>32	>32	>32	16	32	>32	>32
	Gamithromycin	128	>128	>128	>128	16	>128	32	>128
Pleuromutilin	Tiamulin	0.06	0.25	0.06	0.25	0.06	0.25	0.06	0.12
Fluoroquinolone	Enrofloxacin	0.5	1	0.25	1	0.5	2	0.5	1

MIC_50_: the lowest MIC at which at least 50% of the isolates were inhibited in their growth; MIC_90_: the lowest MIC at which at least 90% of the isolates were inhibited in their growth.

## Supplementary Materials

The following are available online https://www.mdpi.com/2079-6382/9/12/882/s1. File S1: Results for gentamicin. File S2: Distribution of MIC values per sector (beef, dairy, veal).
