# Supplementary material for: Antimicrobial Susceptibility of Mycoplasma bovis Isolates from Veal, Dairy and Beef Herds
Source: Antibiotics (Basel). 2020 Dec 9;9(12):882. doi: 10.3390/antibiotics9120882 (PMC7764132; doi:10.3390/antibiotics9120882)
Supplement: Supplementary file 1 [file antibiotics-09-00882-s001.zip › antibiotics-1024940-supplement-File S1 Results of gentamicin.pdf]

antibiotics

MDPI

Article

Antimicrobial Susceptibility of *Mycoplasma bovis* Isolates from Veal, Dairy and Beef Herds

Jade Bokma [1,2,*], Linde Gille [1,†], Koen De Bleecker [3], Jozefien Callens [3], Freddy Haesebrouck [2], Bart Pardon [1,§] and Filip Boyen [2,§]

[1] Department of Large Animal Internal Medicine, Faculty of Veterinary Medicine, Ghent University, Salisburylaan 133, 9820 Merelbeke, Belgium; linde.gille@uliege.be (L.G.); bart.pardon@ugent.be (B.P.)

[2] Department of Pathology, Bacteriology and Avian diseases, Faculty of Veterinary Medicine, Ghent University, Salisburylaan 133, 9820 Merelbeke, Belgium; freddy.haesebrouck@ugent.be (F.H.); filip.boyen@ugent.be (F.B.)

[3] Animal Health Service-Flanders, Industrielaan 29, 8820 Torhout, Belgium; koen.debleecker@dgz.be (K.D.B.); jozefien.callens@dgz.be (J.C.)

* Correspondence: jade.bokma@ugent.be

† Present address: Clinical Department of Production Animals, Faculty of Veterinary Medicine, University of Liège, Quartier Vallée 2 Avenue de Cureghem, 7D, 4000 Liège, Belgium.

§ Bart Pardon and Filip Boyen should be considered joint senior author.

Received: 18 November 2020; Accepted: 8 December 2020; Published: date

Supplementary file 1. Results for gentamicin

For gentamicin the MIC value range of the *M. bovis* PG45 reference strain was 0.5–2 µg/mL. In two previous studies (microbroth and E-test), the MIC for PG45 was 4 µg/mL [12,22]. Unfortunately, gentamicin has not been tested or published very often for *M. bovis* and ranges for the *M. bovis* quality control strains are not described. Also, to the authors knowledge, none of the previous studies testing gentamicin included other quality control strains. In our study, the quality control MIC-values for *Staphylococcus aureus* ATCC 29213 and *Escherichia coli* ATCC 25922 were different from the acceptable quality control ranges as provided by the Sensititre manufacturer. The recommended MICs were 0.12-1 and 0.25-1 µg/mL for *S. aureus* and *E. coli*, respectively, while we observed in general an interval of 4–8 µg/mL (for *S. aureus* 16 µg/mL was observed once). It is likely that specific medium components resulted in an adjusted pH, which probably have altered the results of the quality control strains [28,41]. Nevertheless, considering deviations of both *M. bovis* PG45 reference strain and the quality control strains in this study (and possibly other studies), results of gentamicin should be interpreted with caution.

The results of the MIC determination (Table S1), epidemiological cut-off (Table S2), the distribution over sectors (Table S3), and $MIC_{50/90}$ (Table S4) in this study are shown beneath.

Table S1. Distribution of MIC-values (µg/mL) of 144 M. bovis isolates obtained from cattle in Belgium between 2016–2019.

Class	Antimicrobial	Distribution of MICs (µg/mL)														
		**≤ 0.03**	**≤ 0.06**	**0.12**	**0.25**	**0.5**	**1**	**2**	**4**	**8**	**16**	**32**	**64**	128	>128	Total
Aminoglycoside	Gentamicin	ND				1	10	90	35	3	1		1[a]	ND	ND	141

ND: not determined, [a] MIC ≥ 32; ECOFFs based on the visual estimation method are shown as black vertical lines.

Table S2. Epidemiological cut-offs for *M. bovis* from Belgian cattle (*n* = 144) based on the visual estimation method, NRI and with ECOFFinder, resulting in different percentages of wild type (WT) and non-wild type (n-WT).

Class	Antimicrobial	Visual estimation	WT (%)	n-WT (%)	NRI	WT (%)	n-WT (%)	ECOFFinder (95%/99%) *	WT (%)	n-WT (%)
Aminoglycoside	Gentamicin	>16	99.3	0.7	>8	98.6	1.4	>4/4 (-)	-	-

* plots for residuals were checked and categorized in either well fit (+), poor fit (±) or no fit (-) corresponding to whether the subset values are to believed or not.

Table S3. Results of logistic regression of antimicrobial resistant *M. bovis* isolates obtained from beef, dairy and veal calves between 2016–2019 in Belgium.

		ECOFF	WT (%)	nWT (%)	OR	CI95%	*P* - value
Gentamicin	Beef	> 16	100.0	0.0			
	Dairy	> 16	96.9	3.4			
	Veal	> 16	100.0	0.0			

OR = odds ratio; CI95%: 95% confidence interval.

Table S4. MIC_{50} and MIC_{90} (µg/mL) of all *M. bovis* isolates and per sector, obtained from cattle in Belgium between 2016–2019.

		**Total (*n* = 144)**		**Beef (*n* = 70)**		**Dairy (*n* = 31)**		**Veal (*n* = 32)**	
Class	Antimicrobial	MIC_{50}	MIC_{90}	MIC_{50}	MIC_{90}	MIC_{50}	MIC_{90}	MIC_{50}	MIC_{90}
Aminoglycoside	Gentamicin	2	4	2	4	2	4	2	4

MIC_{50}: the lowest MIC at which at least 50% of the isolates were inhibited in their growth MIC_{90}: the lowest MIC at which at least 90% of the isolates were inhibited in their growth.

References

41. Garrod, L.P.; Waterworth, P.M. Effect of medium composition on the apparent sensitivity of *Pseudomonas aeruginosa* to gentamicin. *J. Clin. Pathol.* **1969**, *22*, 534–538, doi:10.1136/jcp.22.5.534.
